# LTR retrotransposon dynamics in the evolution of the olive (*Olea europaea*) genome

**DOI:** 10.1093/dnares/dsu042

**Published:** 2014-11-26

**Authors:** Elena Barghini, Lucia Natali, Tommaso Giordani, Rosa Maria Cossu, Simone Scalabrin, Federica Cattonaro, Hana Šimková, Jan Vrána, Jaroslav Doležel, Michele Morgante, Andrea Cavallini

**Affiliations:** 1 Department of Agricultural, Food, and Environmental Sciences, University of Pisa, Pisa I-56124, Italy; 2 Institute of Life Sciences, Scuola Superiore Sant'Anna, Pisa, Italy; 3 IGA Technology Service, Udine, Italy; 4 Institute of Experimental Botany, Centre of the Region Haná for Biotechnological and Agricultural Research, Olomouc, Czech Republic; 5 Department of Crop and Environmental Sciences, University of Udine, Udine, Italy; 6 Institute of Applied Genomics, Udine, Italy

**Keywords:** LTR retrotransposons, next-generation sequencing, olive, insertion age, BAC sequencing

## Abstract

Improved knowledge of genome composition, especially of its repetitive component, generates important information for both theoretical and applied research. The olive repetitive component is made up of two main classes of sequences: tandem repeats and retrotransposons (REs). In this study, we provide characterization of a sample of 254 unique full-length long terminal repeat (LTR) REs. In the sample, Ty1-*Copia* elements were more numerous than Ty3-*Gypsy* elements. Mapping a large set of Illumina whole-genome shotgun reads onto the identified retroelement set revealed that *Gypsy* elements are more redundant than *Copia* elements. The insertion time of intact retroelements was estimated based on sister LTR’s divergence. Although some elements inserted relatively recently, the mean insertion age of the isolated retroelements is around 18 million yrs. *Gypsy* and *Copia* retroelements showed different waves of transposition, with *Gypsy* elements especially active between 10 and 25 million yrs ago and nearly inactive in the last 7 million yrs. The occurrence of numerous solo-LTRs related to isolated full-length retroelements was ascertained for two *Gypsy* elements and one *Copia* element. Overall, the results reported in this study show that RE activity (both retrotransposition and DNA loss) has impacted the olive genome structure in more ancient times than in other angiosperms.

## 1. Introduction

The cultivation of olive trees (*Olea europaea* L.) dates back to ancient times. Mythology ascribes the domestication of this species to a divine will: the cultivation of the tree and the treatment of the drupe were taught to the people of Athens by the goddess Athena. In recent years, olive cultivation has been subjected to a growing interest because of the economic, cultural, and ecological importance of olive trees in the Mediterranean area. This interest has grown even outside the Mediterranean area because of health properties of olive oil, related to its composition of fatty acids and secondary metabolites.^1^

From a genetic point of view, this renewed interest in olive tree cultivation has primarily resulted in data on the large genetic variability occurring within *O. europaea* and related species.^2^ Such a large genetic variability can be explained by the prevalent self-incompatibility of olive genotypes, which results in high levels of heterozygosis and DNA polymorphisms. Owing to spontaneous crossings and to the appearance of bud sports over the course of millennia, numerous new genotypes appeared, the best of which were fixed by agamic propagation.^3^

*Olea europaea* L. has a medium-sized haploid genome of 1.4 Gb,^4^ whose structure has been long uncharacterized. Concerning the repetitive component of the genome, before 2014 only four tandem repeat sequences had been characterized and localized by cytological hybridization on chromosomes.^5–7^ In addition, a few putative retrotransposon (RE) fragments were isolated and sequenced.^8,9^

After sequencing the whole genome of some plant species, the knowledge of the structure and organization of plant genomes has been substantially improved, leading to the view that genome evolution of angiosperms has been accompanied—and possibly promoted—by polyploidization events and differential amplification of repetitive DNA.^10^

The repetitive DNA in plants is mainly represented by Class I transposons (REs) that are capable of replicating through a ‘copy and paste’ mechanism and can potentially increase the genome size of their host species in a very short time.^11^

Among REs, elements that contain direct long terminal repeats (LTRs) are predominant in plants. LTR-REs vary in size from a few hundred base pairs to over 10 kb, with LTRs that usually contain the promoter and RNA processing signals.^12^ Internal to the 5′ and 3′ LTRs, respectively, are the primer-binding site (PBS) and the polypurine tract (PPT), which provide the signals for reverse transcription of RE transcripts into the cDNA that will be reintegrated into the genome. In autonomous elements, these two sequence sites flank the region that contains ORFs for Gag, a structural protein of the virus-like particles, and for Pol. Pol encodes a polyprotein with protease, reverse transcriptase (RT), RNaseH, and integrase enzyme domains, which are required for the replication and the integration of the elements in the host chromosomes.

LTR-REs belong to two major superfamilies, called *Gypsy* and *Copia*, differing in the position of the integrase domain within the encoded polyprotein.

The occurrence of different RE families, characterized by sequence variability in both the coding, transcribed portion and the LTRs^13^ has been reported in several genomes. These families were probably generated by the replicative mechanism of LTR-REs, coupled with the error-prone nature of transcription and reverse transcription. RE families have amplified differentially in different lineages within plant genera or even within a single species (for example, in maize) over a time-span of <1 million yrs (MY).^14^ Similar events have taken place in several cereal species: for example, in the genome of *Sorghum bicolor*, the insertion of transposable elements and their removal by unequal recombination or by DNA loss resulted in an average RE insertion age of 0.8 MY, with 50% of the detected elements having inserted within the last 500,000 yrs.^15^ Such processes have also been detected in some dicots, although to a less dramatic extent.^11,16,17^

Recently, next-generation sequencing technologies and different computational procedures have been used to gain a general insight into the composition of the olive genome and its repetitive fraction.^18^ Illumina and 454 reads from genomic DNA were assembled following different procedures, obtaining >200,000 differently redundant contigs, with a mean length of >1,000 nt. By combining identification and mapping of repeated sequences, it was established that tandem repeats represent a very large portion of the olive genome (∼31%), consisting of six main families of different length. The other large redundant class in the olive genome is represented by transposable elements, especially LTR-REs.^18^

The identification and characterization of olive LTR-REs were, however, difficult because of the lack of large sequenced genomic regions. For example, an accurate dating of amplification events of the LTR-RE component requires a comparison of the two LTR sequences from individual, full-length elements.^19^

In the frame of a project aimed to sequence the olive genome, a bacterial artificial chromosome (BAC) library was produced. A number of BAC clones were pooled and then sequenced using the Illumina procedure. These sequences were assembled and the resulting contigs analysed to identify full-length LTR-REs, allowing the first characterization of such elements in olive, especially in relation to their insertion age.

## 2. Materials and methods

### 2.1. Sequencing of BAC clones

A BAC library from *O. europaea* cv. Leccino was produced as follows. Cell nuclei were isolated from the youngest leaves of the olive tree following the protocol of Doležel *et al.*^20^ Briefly, the leaves were fixed for 20 min at 5°C in 2% (v/v) formaldehyde and immediately afterwards chopped by a razor blade in ice-cold isolation buffer (15 mM Tris, 10 mM EDTA, 130 mM KCl, 20 mM NaCl, 1 mM spermine, 1 mM spermidine, 45 mM β-mercaptoethanol, and 0.1% Triton X-100, pH 9.4). The suspension of released nuclei was passed through a 50-µm-pore nylon mesh to remove large tissue and cellular fragments, and was then stained using DAPI (2 µg/ml). Intact nuclei were sorted using flow cytometry and used to prepare high-molecular-weight (HMW) DNA.^21^ HMW DNA of 1.8 million nuclei (∼5.4 µg DNA) was used to construct a large insert library cloned in pIndigoBAC-5 vector (Epicentre, Madison, WI, USA) as previously described.^22^

We shotgun-sequenced 12 pools each formed by 384 BACs (corresponding to 12,384-well library plates) for a total of 4,608 clones using the Illumina procedure. DNA from each plate pool was prepared using the Illustra TempliPhi Large Construct V2 kit (Resnova). Each BAC pool was then individually assembled as described below.

Paired-end libraries were prepared by using the Nextera DNA Sample Prep Kit (Illumina, Inc., San Diego, CA, USA) and sequenced in two lanes on an Illumina HiSeq2000 at the 12-plex level of multiplexing, producing from 30 to 50 million reads per pool (paired-end 100-nt set-up). The processing of fluorescent images into sequences, base-calling, and quality value calculations was performed using the Illumina data-processing pipeline (version 1.8.2).

Illumina reads were then processed to remove adapters using Cutadapt (https://pypi.python.org/pypi/cutadapt/1.4.2, 12 November 2014, date last accessed),^23^ with default parameters except -O 10 -n 2 -m 50 and finally paired again using an internally developed Python script. To trim low-quality regions and to remove bacterial contaminants and olive chloroplast sequence, reads were further processed with ERNE-FILTER (http://erne.sourceforge.net, 12 November 2014, date last accessed) using default parameters except–min-size 50 and —errors-rate 25.

Illumina reads from each individual BAC pool were assembled using ABySS^24^ with the following parameters: *k* = 71, l = 1, aligner = map, *b* = 1,000,000, *P* = 0.95, *s* = 500, *n* = 10. An internally developed Perl script was used to remove scaffolds shorter than 500 nt.

### 2.2. Identification of full-length LTR-REs

Assembled contigs longer than 10,000 nt were surveyed for the identification of full-length LTR-REs based on structural features and sequence similarity to the *Olea* RE database^18^ and to public sequence databases (non-redundant nucleotide and protein NCBI databases, RepBase database).

Structural features were identified using the LTR-FINDER^25^ and DOTTER^26^ software. Alignment boundaries were obtained by adjusting the ends of LTR-pair candidates using the Smith–Waterman algorithm. These boundaries were re-adjusted based on the occurrence of typical LTR-RE features that include the following: being flanked by the dinucleotides TG and CA at 5′ and 3′ ends, respectively; the presence of a target-site duplication (TSD) of 4–6 nt; a putative 15- to 18-nt PBS, complementary to a tRNA at the end of the putative 5′-LTR; and a 20- to 25-nt PPT just upstream of the 5′ end of the 3′ LTR.

All putative LTR-REs were subsequently annotated by BLASTX and BLASTN against the public non-redundant databases at NCBI, and by RepeatMasker against the RepeatExplorer-based database of olive-repeated sequences.^18^ To limit false-positive detection, we used a fixed *E*-value threshold of *E* < 10^−5^ for BLASTN and *E* < 10^−10^ for BLASTX. The full-length REs that were identified as belonging to *Gypsy* or *Copia* superfamilies were then used as a reference database for a further BLASTN search, in order to classify previously unclassified elements.

In other analyses, 10,000 nt upstream and downstream of each LTR-RE was subjected, whenever possible, to a BLAST search to identify other sequences (coding and/or non-coding) occurring in proximity of each RE.

### 2.3. Estimation of LTR-RE abundance

To estimate the redundancy of the LTR-RE set and of the *Gypsy* and *Copia* superfamilies, a large set of Illumina whole-genome shotgun reads (total coverage 8.1×)^18^ was mapped onto all isolated elements, using CLC-BIO Genomic Workbench 6.5.1, with the following parameters: mismatch cost = 1, deletion cost = 1, insertion cost = 1, similarity = 0.9, and length fraction = 0.9. To obtain reads of constant length, all bases exceeding 75 nt were cut. In this analysis, multireads (i.e. those reads that matched multiple distinct sequences) were distributed randomly, and hence, the number of mapped reads to a single sequence would be only an indication of its redundancy. On the other hand, if all sequences of a sequence class are taken together, the total number of mapped reads (in respect to total genomic reads) reveals the effective redundancy of that class.

The redundancy level of each single sequence was estimated by mapping the same large set of Illumina whole-genome shotgun reads as above onto each isolated RE, one by one. Redundancy values are reported as the number of mapped reads per kb of sequence length.

In other analyses, in order to evaluate the occurrence of solo-LTRs, the same read set was mapped onto each isolated RE one by one, keeping the 5′-LTR and the inter-LTR region separated.

### 2.4. Phylogenetic analyses

Full-length LTR-REs were scored for retrotranscriptase, RNAseH, and integrase domains of *Gypsy* and *Copia* elements—separately—using TBLASTN against an internally developed library of RE proteins. All sequences of at least 80 amino acids were collected and aligned using CLUSTALW,^27^ and a tree was generated. The tree was visualized using FigTree (http://tree.bio.ed.ac.uk/software/figtree/, 12 November 2014, date last accessed).

### 2.5. Insertion age calculation of full-length LTR-REs

RE insertion age was estimated comparing the 5′- and 3′-LTRs of each putative RE. The two LTRs of a single RE are identical at the time of insertion because they are copied from the same template.

A synonymous substitution rate was calculated comparing 20 protein-coding sequences (longer than 200 nt, putatively nuclear, and unique) of another species belonging to the *Oleaceae* family, *Fraxinus excelsior* (The British Ash Tree Genome Project, http://www.ashgenome.org/, 12 November 2014, date last accessed) to orthologous sequences of *O. europaea*, selected from an available olive transcriptome.^28^ Rates of synonymous nucleotide substitution for each gene sequence were calculated by the method of Nei and Gojobori^29^ with the Jukes–Cantor correction as implemented in the DnaSP program.^30^ As the estimated separation between *Olea* and *Fraxinus* is dated to between 40 and 45 MY ago,^31^ 42.5 MY was used for estimating the synonymous nucleotide substitution rate.

The two LTRs of each full-length RE were aligned with the ClustalW software,^26^ indels were eliminated, and the number of synonymous nucleotide substitutions per site was calculated using an internally developed pipeline. As proposed by Ma and Bennetzen,^32^ we used twice the mean number of synonymous substitutions per site per year as the nucleotide substitution rate between LTRs. Based on this rate, the insertion time for each full-length RE was estimated.

## 3. Results

### 3.1. Isolation and annotation of full-length LTR-REs

The BAC library consisted of 44,928 clones with an average insert size of 112 kb. Considering the 1C genome size of *O. europaea* cv. Leccino (1.49 Gb, determined by DNA flow cytometry; J. Čížková, personal communication), the library should represent 3.3 genome equivalents.

We sequenced a total of 4,608 clones in pools of 384 and, after assembly of each individual pool, we selected all contigs longer than 10 kb (275, for a total of 7,653,690 nt). These were scored for the occurrence of putative full-length LTR-REs, searching for structural features and sequence similarities, that is, the occurrence of two relatively intact LTRs, of identified PPT and PBS sites, and of flanking TSDs.

A set of 254 putative full-length REs was isolated from 245 over 275 contigs (1 RE from each of 236 contigs and 2 REs from each of 9 contigs). We defined full-length elements based on the occurrence of intact ends, irrespective of whether these elements were potentially functional or contained inactivating mutations in their internal sequence (Table 1). Many sequences showed all distinctive structural features of REs, although in some cases one feature was missing.

**Table 1.** Mean characteristics of *Copia*, *Gypsy*, and unknown putative full-length retroelements identified in the BAC clones

Superfamily	No. of REs	RE length (nt) ± SE	5′ LTR length (nt) ± SE	3′ LTR length (nt) ± SE	Mean number of mapped reads per 1,000 nt ± SE	Putative insertion age (MY) ± SE
*Copia*	166	5,605.0 ± 151.9	564.7 ± 41.0	540.1 ± 28.1	6,322.3 ± 708.7	17.13 ± 0.72
*Gypsy*	81	7,632.0 ± 348.3	643.2 ± 45.5	646.1 ± 46.4	8,749.6 ± 1,049.0	19.34 ± 0.77
Unknown	7	4,705.0 ± 2,764.0	464.4 ± 170.3	458.4 ± 164.8	1,477.0 ± 965.0	21.10 ± 3.39

The isolated REs covered a total of 1,584,566 nt over 7,653,690 nt, with a mean length of 6226.5 nt. They were classified as *Gypsy* or *Copia* according to BLAST searches against NCBI, RepBase,^33^ and OLEAREP^18^ databases and to a subsequent BLAST search using the same olive REs as a reference database.

The majority of isolated full-length REs belonged to the *Copia* superfamily (166), followed by the *Gypsy* superfamily (81, of which 36 contained an integrase chromodomain^34^). Seven REs were classified as unknown because they lacked distinctive protein-coding sequences suitable for classifying the element. For 222, 171, and 253 elements, the putative TSD, PBS, and PPT were identified, respectively. Seventy-two elements showed all typical protein domains of LTR-REs. The main features of each isolated RE are reported in Supplementary Data S1.

Sequences adjacent to isolated REs were also scored for similarity to other genic or non-genic sequences. The occurrence of putative genes and transposon-related sequences is summarized in Table 2. In many cases (99 REs, 38.93%), the isolated full-length REs lied in proximity of gene sequences. Ninety-five REs (37.40%) were close to REs or DNA transposon fragments. Only 16 REs (6.30%) were flanked at both sides by transposon fragments, possibly representing loci with nested elements.

**Table 2.** Occurrence of sequences belonging to genes and/or transposable elements (REs and DNA transposons) in the upstream and/or downstream regions of isolated full-length REs

Upstream sequence	Downstream sequence	No. of full-length REs
Gene	Gene	13 (5.12%)
Gene	Unclassified	41 (16.14%)
Unclassified	Gene	27 (10.63%)
Gene	Transposon	5 (1.97%)
Transposon	Gene	13 (5.12%)
Transposon	Unclassified	36 (14.17%)
Unclassified	Transposon	25 (9.84%)
Transposon	Transposon	16 (6.30%)
Unclassified	Unclassified	78 (30.71%)

### 3.2. Genomic redundancy of isolated LTR-REs

Considering the percentage of Illumina reads that matched to a class of sequences as an indicator of the proportion of that class in the olive genome, previous experiments^18^ indicated that LTR-REs account for 38.8% of the olive genome. Using the same set of reads for mapping the 254 REs isolated from BAC clones, we found that they were mapped by 17,107,830 reads, corresponding to 12.3% of the genome, that is, these REs represent around one-third of the RE population in the genome.

**Figure 1.**
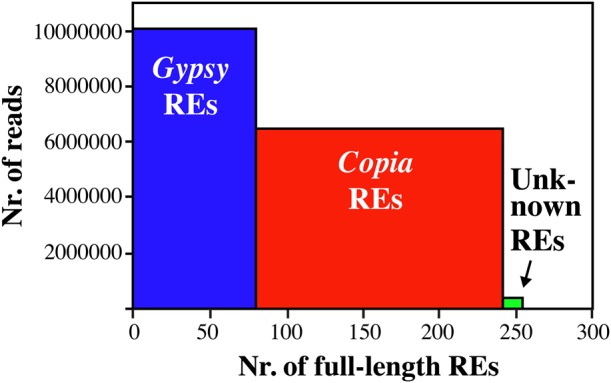
Number of full-length REs identified in this study, separated according to their superfamily. Each bar in the histogram shows the number of Illumina reads that matched to all REs (height) and the number of REs (width) of each superfamily.

Mapping results of the different RE superfamilies are summarized in Fig. 1. In a previous work on olive genome,^18^ the ratio between redundancy of *Gypsy* and *Copia* REs was 1.17. In the RE sample described here, the ratio is quite different, amounting to 0.92, indicating that the REs identified in this study do not represent the whole olive RE set. However, it is worth noting that, although *Gypsy* full-length elements are only 81 versus 166 *Copia* elements, they are mapped by a number of reads similar to that mapping to the *Copia* REs. This result confirms that, in the olive genome, the number of *Gypsy* families is lower than that of *Copia*, but *Gypsy* REs are more redundant than *Copia* REs.^18^

**Figure 2.**
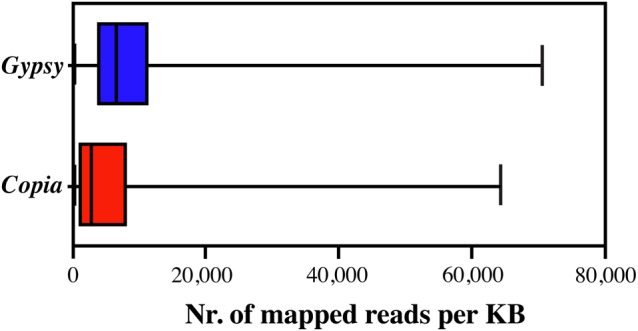
Box and whiskers plot of RE redundancy (calculated as the number of mapped reads per kb) of olive *Copia* and *Gypsy* REs. The boxes represent the 25–75%, whiskers represent the whole range of values, and lines in the box represent the mean values of the distribution.

The distribution of the number of mapped reads of isolated *Gypsy* and *Copia* REs is reported in Fig. 2. *Gypsy* elements showed a larger mean and distribution around the mean compared with *Copia*.

**Figure 3.**
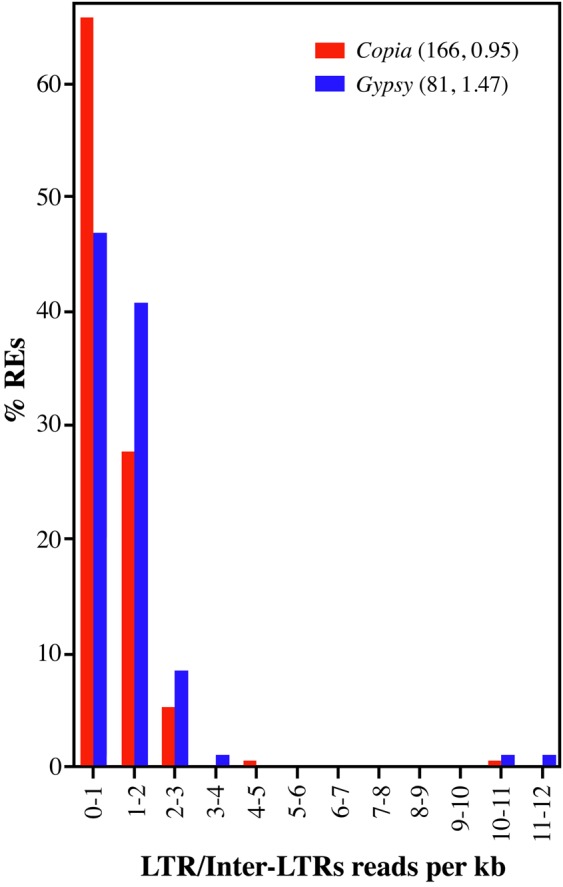
Distribution of full-length olive LTR-REs according to the ratio between the number of mapped reads per kb measured separately on LTR and inter-LTR regions.

To estimate the equilibrium between RE replication and RE loss, Illumina reads were mapped onto each full-length LTR-RE, keeping LTR sequences separate from the respective inter-LTR region. The results of this analysis are shown in Fig. 3.

The ratio between the numbers of mapped reads per kb between the 5′-LTR and the respective inter-LTR DNA sequence ranged from 0.003 to 20.52. If all REs belonging to the same family were intact, that is, composed of two LTRs and one inter-LTR region, the ratio should have been 2. Conservatively, we considered the occurrence of solo-LTRs only for those LTR-REs whose ratio was higher than 2.5. Only a small number of REs showed ratios higher than 2.5 (16 of 254—eight *Gypsy*, seven *Copia*, and one unknown element), and only three showed ratios higher than 10.

Many LTR-REs showed a ratio lower than 2 (Fig. 3), that is, the inter-LTR region was more represented in the genome than in the LTR. This result suggests the presence of different families that share, at least in part, the inter-LTR region and show a higher level of sequence conservation of the *pol* protein-coding domains. Interestingly, Fig. 3 suggests that this aspect is especially true for *Copia* REs.

Finally, one *Copia* and two *Gypsy* elements showed the highest ratio between the numbers of mapped reads per kb of the LTR and inter-LTRs (Fig. 3), indicating that unequal homologous recombination has particularly affected elements similar to these two REs. The *Copia* RE shows sequence similarity to *Ale* retroelements of other species and it is medium redundant. Both *Gypsy* REs show similarity to chromodomain-containing Res, and the estimated redundancy of the full-length forms of them is very high.

### 3.3. Phylogenetic relationships among isolated LTR-REs

**Figure 4.**
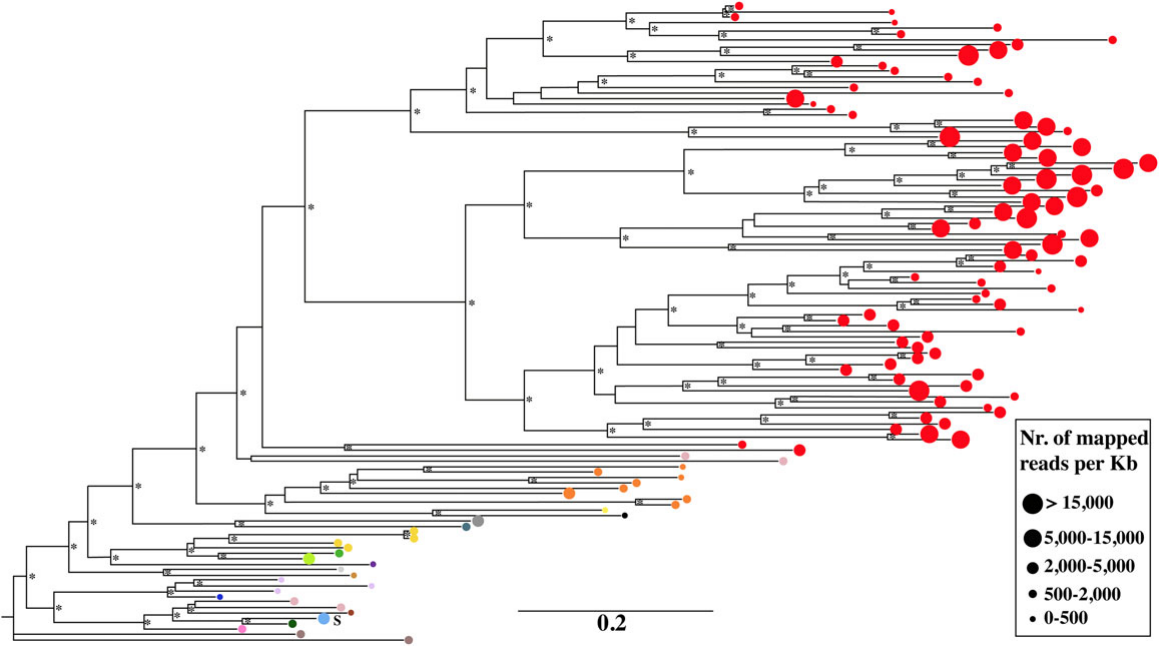
Phylogenetic tree obtained from the neighbour-joining analysis of 93 *Copia* retrotranscriptase sequences. Different *Copia* families are indicated by different grey tones (different colours in the online version of DNA Research). For each RE, the area of the symbol indicates the redundancy of that element in the olive genome. The bar represents the genetic distance. Asterisks indicate bootstrap values >50%. The letter S indicates a *Copia* RE with an LTR/inter-LTR ratio of >10.

**Figure 5.**
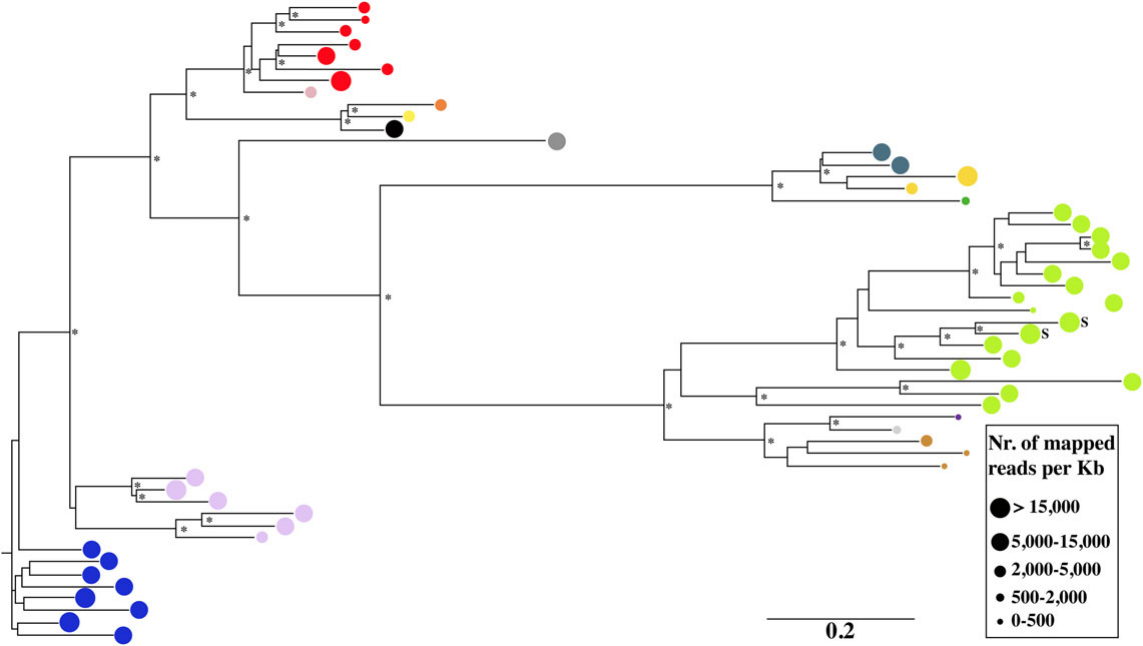
Phylogenetic tree obtained from the neighbour-joining analysis of 43 *Gypsy* retrotranscriptase sequences. Different *Gypsy* families are indicated by different grey tones (different colours in the online version of DNA Research). For each RE, the area of the symbol indicates the redundancy of that element in the olive genome. The bar represents the genetic distance. Asterisks indicate bootstrap values >50%. The letter S indicates two *Gypsy* REs with an LTR/inter-LTR ratio of >10.

Phylogenetic analyses were performed by the neighbour-joining method to evaluate the relationship between isolated LTR-REs. Two phylogenetic trees were constructed based on the transcribed putative retrotranscriptase sequence of 93 *Copia* and 43 *Gypsy* REs (Figs 4 and 5). The bootstrap values suggest the occurrence of distinct families, indicated in the figures by different colours.

The redundancy of each element is also reported in Figs 4 and 5. Within each family, especially for the *Gypsy* superfamily, different elements often showed similar redundancy. In many cases, the least redundant *Copia* elements did not cluster in the tree. In contrast, *Gypsy* unclustered elements were generally highly redundant. It should be noted that 9 of 20 *Copia* elements with mapped reads/kb >15,000 are reported in the retrotranscriptase tree, all belonging to the same, large cluster. In the other cases, they did not carry the RT-encoding domain. Using other RE protein domains, seven more *Copia* elements with mapped reads/kb >15,000 resulted in the same cluster (data not shown), indicating that the most redundant elements of this superfamily share some sequence similarity. Four of 20 most redundant REs did not carry protein domains, hence their relationship to the other sequences remained unknown.

### 3.4. Putative insertion dates of LTR-REs

Intact retroelements have a built-in molecular clock that is useful for estimating their insertion times, based on sister LTR divergence. In fact, when an RE is inserted into the genome, its LTRs are usually 100% identical. Considering that the retroelement transcription starts from the R region in the 5′-LTR and terminates at the end of the R region in the 3′-LTR (thus including only one copy of each U5 and U3 regions), the combination of single copy U5 and U3 regions with a hybrid R region during reverse transcription into cDNA yields two identical LTRs at both ends, prior to integration.^12^ Mutations then occur within the two LTRs, and as more time passes since the insertion, the larger the genetic distance between LTRs becomes. Hence, the RE insertion time can be estimated using a nucleotide substitution rate suitable for such elements, which is assumed to be higher than that of gene regions.^32^

We estimated the synonymous substitution rate of genes in *O. europaea* by comparing orthologous cDNA sequences of *O. europaea* and *F. excelsior* (a species belonging to the Oleaceae family, for which a large amount of sequence data are available; Buggs and Sollars, personal communication), that is, 20 coding sequences for a total of 7,282 nt in *O. europaea* and 6,552 nt in *Fraxinus* (Supplementary Table S1). The mean number of synonymous substitutions per site (*K*_s_) was 0.077.

Based on the separation between the olive and the ash tree, estimated to be ∼42.5 MY,^31^ the resulting synonymous substitution rate is 1.8 × 10^−9^, which is lower than those reported for herbaceous species, and similar to those of other perennial tree species.^17,35^ In fact, it is known that the generation time of a species affects its nucleotide substitution rate^36^ and trees have a much longer generation time than herbaceous species.

It was proposed that mutation rates for LTR-REs may be approximately 2-fold higher than synonymous substitution rates for protein-coding genes.^32^ Consequently, in our calculations of LTR-RE insertion dates, we used a substitution rate per year of 3.6 × 10^−9^.

**Figure 6.**
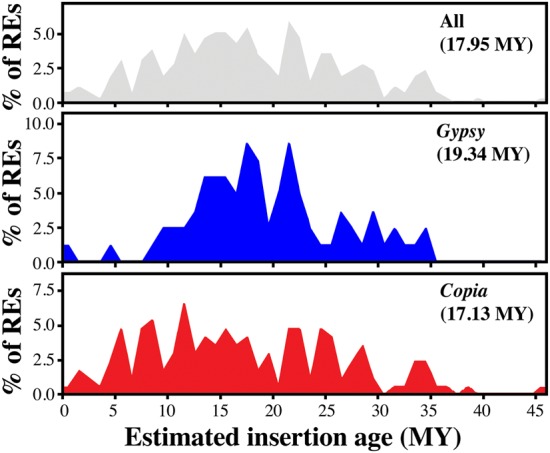
Distributions of full-length REs identified in this study, according to their estimated insertion ages (MY). Mean insertion dates are reported in parentheses.

The sequences of LTR pairs were compared and the putative insertion times were calculated for each full-length LTR-RE. Taking into account the whole set of full-length REs, the nucleotide distance (*K*_s_) between sister LTRs showed a large variation between retroelements, representing a time-span of, at most, 46 MY. The putative mean age of analysed LTR-REs was 17.94 MY (standard error = 0.54 MY). The distribution of full-length LTR-REs according to their putative insertion date is reported in Fig. 6.

Analysis of the insertion date profiles provides evidence for a partial overlap among retrotransposition waves of *Gypsy* and *Copia* full-length LTR-REs. For example, taking into consideration the last 7 MY, nearly all retrotransposition events involve *Copia* elements. On the other hand, *Gypsy*-related elements show a large peak of retrotransposition between 10 and 25 MY ago, in contrast to *Copia* REs, whose retrotransposition activity is scattered over the last 40 MY.

Interestingly, two REs (one *Copia* and one *Gypsy*) did not show variations between their LTRs, suggesting that insertion should have occurred between 0 and 1.760 MY ago and between 0 and 0.387 MY ago, respectively, that is, the retrotransposition process could still be active.

**Figure 7.**
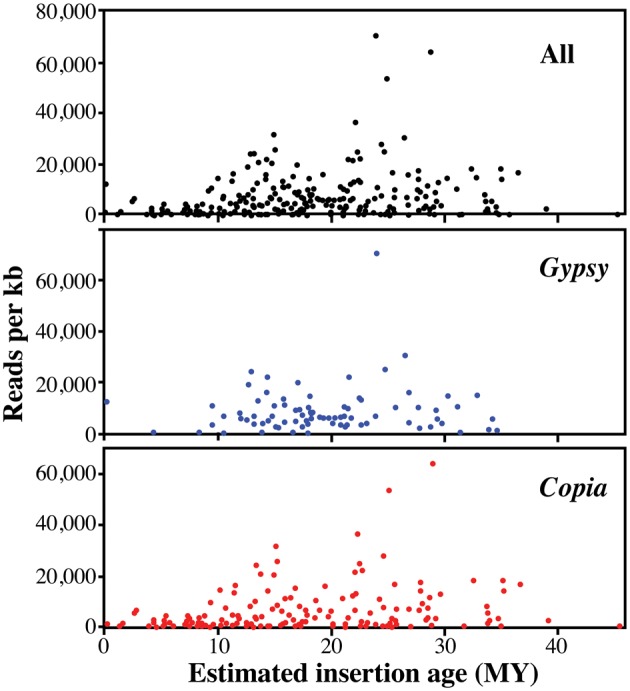
The relationship between estimated insertion ages (MY) and the redundancy of full-length REs identified in this study.

The relationship between the insertion age of an RE and its redundancy in the genome is shown in Fig. 7, and indicates the intensity of retrotransposition activity in the period in which retrotransposition has occurred, although the case that the insertion time of an element cannot correspond to the period of largest activity of its RE family cannot be ruled out.

Figure 7 suggests that intense retrotransposition waves have occurred, for some RE families of both superfamilies, between 10 and 30 MY ago. One *Gypsy* and two *Copia* families may have been especially active between 23 and 29 MY ago.

## 4. Discussion

The repetitive component of the olive genome amounts to >70%.^18^ Around 30% of the genome is made of tandem repeat sequences and 40% of transposable elements. Such a genome composition appears to be peculiar to *O. europaea*. In other species, tandem repeats account for <10% of the genome, with the exception of the genome of cucumber,^37^ which is composed of ∼23% of such sequences, a percentage, however, lower than that measured in the olive genome. In plant species other than olive, transposable elements account for the vast majority of the repetitive component. The partial replacement of REs by tandem repeats in the olive genome prompted us to study the dynamics of LTR-REs in this species.

We analysed olive LTR-REs based on sister LTR identification in large contigs obtained assembling Illumina-sequenced BAC pools. By this approach, only putative full-length REs, that is, with two very similar LTRs, were scored. In total, we have isolated 254 full-length LTR-REs that can be added to the already available RE sequences.^18^

The isolated full-length REs and their remnants represent 12.33% of the olive genome and constitute a particular fraction of these sequences. First, the sample of REs may have been biased by the assembly process. Repetitive elements represent the most difficult parts to assemble in any shotgun assembly project and are often under-represented in whole-genome assemblies. We assembled pools of 384 BACs that should on average each comprise 42,000 nt of genomic sequence, corresponding to <3% of the total genome size for olive. This should make the assembly of repetitive elements easier, even though difficulties may remain for high-copy sequences (that are represented in multiple copies within each pool) as well as for elements that have identical LTRs at their ends, where it may have been difficult to assemble a complete element with both LTRs. This may have affected not only the representation of different families and superfamilies, but also our estimation of insertion times of elements, because the REs with identical or nearly identical LTRs may not have been reconstructed as complete elements. Secondly, the method used to isolate full-length REs allowed us to select especially dispersed elements, whereas nested elements were probably under-represented in the sample, and it is known that nested REs constitute a large fraction of the repetitive component in medium- to large-sized genomes, such as those of maize^38^ or sunflower.^16^ It is possible that the REs isolated in this work are preferentially located in the gene-rich fraction of the olive genome, as suggested by the large number of protein-coding sequences that were found in the upstream or downstream region of REs. Nevertheless, the selected set of 254 full-length REs constitutes a valuable sample of these sequences, allowing an analysis of RE dynamics in this species.

In angiosperms, *Gypsy* and *Copia* superfamilies are differently represented in the genome. Different ratios between *Gypsy* and *Copia* RE frequencies were reported,^39^ ranging from 5 : 1 in papaya^40^ to 1 : 2 in grapevine.^41^ Analysis of the whole olive genome showed a ratio of 1.17.^18^ The isolated olive full-length REs showed, on the contrary, a slight prevalence of *Copia* over *Gypsy* elements. This could be related to a different accumulation in genic and intergenic regions of the genome between *Copia* and *Gypsy* elements. In fact, *Copia* REs are often scattered on the chromosomes, whereas *Gypsy* elements preferentially accumulate—in a nested way—in specific chromosomal locations and structures, such as centromeric and pericentromeric regions.^42^ In this sense, the larger number of isolated *Copia* REs would be a consequence of the procedure used for RE identification. On the other hand, analysis of RE redundancy clearly showed that isolated full-length *Gypsy* elements, although at a lower number than *Copia*, account for a larger fraction of the genome, suggesting that they are more redundant, and confirming the previously reported results.^18^

The occurrence of RE families in the olive genome was established according primarily to sequence similarity of their RT-coding sequences. Although, in many cases, bootstrap values were <50%, the trees suggest the occurrence of a number of RE clusters. It is to be noted that the most redundant elements (both *Gypsy* and *Copia*) are clustered, indicating that the corresponding families have been highly active during olive genome evolution.

The relatively low frequency of REs in the olive genome could be related to a low rate of retrotransposition, but also to RE loss.^43^ RE DNA removal is driven in plants by a number of mechanisms, including DNA rearrangements and unequal homologous recombination; solo-LTRs are the main products of such processes.^32,44,45^

Analysing the relative redundancy of LTRs and inter-LTR regions in the same full-length RE allowed us to evaluate the occurrence of solo-LTRs related (i.e. belonging to the same family) to that RE. Solo-LTRs related to the isolated full-length REs were rare: only 16 of 254 REs showed a ratio between the number of mapped reads per kb of LTR and inter-LTR >2.5. The LTR/inter-LTR ratios of *Gypsy* elements were generally higher than those of *Copia*. These ratios were especially high for one *Copia* and two *Gypsy* elements, sharing sequence similarity with chromodomain-containing and *Ale* REs of other species, indicating the occurrence of a large number of solo-LTRs for RE families that are related to these full-length elements. However, the presence of REs sharing LTRs but not internal regions cannot be ruled out and could lead to an overestimation of solo-LTR frequencies.

If genome size derives from an equilibrium between enlargement (by polyploidization and RE amplification) and reduction (by DNA loss) in olive, the genome size was increased by massive amplification of REs and of tandem repeats.^18^ This increase was partly counterbalanced by DNA loss, related to both *Gypsy* and *Copia* elements, in contrast to other species in which solo-LTRs have been found especially in *Copia* elements, as, for example, in sunflower.^46,47^

Concerning the amplification of REs, the identification of sister LTRs allowed us, for the first time, to date the insertion of REs in the olive genome, using the method established by SanMiguel *et al.*^19^ in maize. Obviously, the estimation of insertion time by the number of mutations in sister LTRs is subject to error, because it assumes that the same mutation rates operate in all retroelements and chromosome positions, while that was not proved to be true in, for example, the genus *Oryza*.^48^ However, this method appears as the most suitable to study RE dynamics.

Analysis of sister LTR similarity indicates that, in olive, both *Gypsy* and *Copia* REs have been active in the same period. Nearly, all the identified full-length elements appear to be mobilized in a time-span of 40 MY (Fig. 4), although it is conceivable that more ancient REs are not easily recognizable because of accumulation of variability between sister LTRs.

The mean insertion date of olive *Copia* full-length REs is lower than that of *Gypsy*. The insertion date profiles indicate that, during the last 40 MY, *Copia* and *Gypsy* REs have both been active, but with different time courses. For example, only one isolated *Gypsy* full-length RE inserted between 1 and 8 MY ago. Moreover, the percentage of *Gypsy* REs inserted between 10 and 25 MY ago, and hence, presumably, their retrotransposition activity is by far larger than that of *Copia* elements. Different amplification histories of these RE superfamilies during the evolution of the host species have been reported in many plant species. *De facto* analysis of crop genomes in a phylogenetic context reveals scarce congruence in RE content and highlights differences in the success of different RE types.^39^

In contrast to other species, such as maize^14^ and sunflower,^49,50^ in which the RE burst is very recent and probably still occurring, in the olive genome the insertion of new REs appears to be decreasing in frequency in the last 8 MY, for both *Gypsy* and *Copia* REs. A similar time course of the RE amplification wave was reported in the genome of a gymnosperm, the Norway spruce.^35^ While our estimates of insertion ages may have been biased against the most recent elements by the assembly process as discussed previously, it is to be considered that all those REs interrupted by other elements (i.e. presumably older than inserted ones) are not included in the sample. The observation of a considerable number of elements inserted >10 MY ago is still valid and represents a clear distinctive feature of the olive genome in comparison with other angiosperm genomes analysed so far.

The relationship between insertion time of an element and its redundancy offers further insights into olive RE dynamics. *Gypsy* and *Copia* full-length REs inserted between 10 and 30 MY ago are by far the most abundant, while recently inserted elements show low levels of redundancy. This is expected because it is known that lowly redundant elements are more prone to escape RE silencing. The age distribution of REs further suggests a progressive reduction in RE activity from 20 MY ago until now.

Dating the amplification process of another major repeat class in the olive genome, the tandem repeats, could clarify if a type of ‘competition’ in the genome colonization has occurred between repeat types during olive evolution.

Concerning olive RE transcription, no data are currently available. Ancient LTR-REs are generally inactive or less active than young ones, probably because of the accumulation of mutations determining premature stop codons in the coding portion of the LTR-RE, as observed in rice.^51^ Moreover, there is also a strong control of RE activity by the host species; it has been established that REs are especially silenced by siRNA.^52^ It is plausible that the large number of LTR-RE fragments spread throughout the olive genome can produce siRNAs that silence related retroelements.

In conclusion, our analyses show many aspects of RE dynamics in the evolution of the olive genome. Some data are similar to those observed in other plant species, but some peculiarities of the olive genome also emerged for this repeat class, besides the extreme redundancy of tandem repeats. All these data support the theory that if RE dynamics are similar, including birth through transposition, silencing and then death by both random mutation and possibly deletion from the genome,^53^ the factors inducing such dynamics might be different in different RE lineages and possibly related to the ‘ecosystem’ in which the REs interact and compete.^54^ Hence, according to Venner *et al.*,^55^ olive REs can be considered a community of different organisms in the genome, with ‘species’ (corresponding to RE superfamilies) and ‘subspecies’, characterized by different LTR sequences, activity, and evolution history.

## 5. Data retrieval

Whole-genome shotgun sequences described are available on NCBI Sequence Read Archive under SRA Project number SRX465835. Assembled BAC sequences and RE sequences are available at the Sequence Repository Page of the Department of Agriculture, Food, and Environment of University of Pisa (http://www.agr.unipi.it/ricerca/plant-genetics-and-genomics-lab/sequence-repository.html, 12 November 2014, date last accessed) and on the NCBI website (http://www.ncbi.nlm.nih.gov/, 12 November 2014, date last accessed) under the accession numbers KM577349–KM577602.

## Supplementary data

Supplementary data are available at www.dnaresearch.oxfordjournals.org.

## Funding

This research work was funded by Ministero delle Politiche Agricole, Italy, Progetto ‘OLEA: Genomica e miglioramento genetico dell'olivo’. BAC library construction was supported by the Czech Science Foundation (award no. P501/12/G090) and by grant award LO1204 from the National Program of Sustainability I. Funding to pay the Open Access publication charges for this article was provided by Department of Agriculture, Food and Environment, University of Pisa.

## Supplementary Material

Supplementary Data
