# Supplementary material for: LTR retrotransposon dynamics in the evolution of the olive (Olea europaea) genome
Source: DNA Res. 2014 Nov 26;22(1):91–100. doi: 10.1093/dnares/dsu042 (PMC4379980; doi:10.1093/dnares/dsu042)
Supplement: Supplementary Data [file supp_22_1_91__index.html]

LTR retrotransposon dynamics in the evolution of the olive (Olea europaea) genome — Supplementary Data 

# LTR retrotransposon dynamics in the evolution of the olive (*Olea europaea*) genome

## Supplementary Data

Supplementary Data

**Files in this Data Supplement:**

- Supplementary Data - Doc file
- Supplementary Table 1 - xlsx file
