# Supplementary material for: LTR retrotransposon dynamics in the evolution of the olive (Olea europaea) genome
Source: DNA Res. 2014 Nov 26;22(1):91–100. doi: 10.1093/dnares/dsu042 (PMC4379980; doi:10.1093/dnares/dsu042)
Supplement: Supplementary Data [file supp_dsu042_dsu042supp.doc]

**Supplementary Table S1.** Number of overlapping sites and number of synonymous substitutions per site (K_s_) in 20 putatively orthologous gene sequences of *O. europaea* and *Fraxinus excelsior.*

| Putative function | Number of sites | K_s_ |
| --- | --- | --- |
| Molybdopterin molybdenum transferase | 241 | 0.0610 |
| U4/U6 sn ribonucleoprotein Prp3-like | 307 | 0.0446 |
| Phosphatidylinositol-glycan biosynthesis class S protein | 276 | 0.0571 |
| Rab GTPase interacting factor | 347 | 0.1089 |
| Glucan endo-1,3-beta-glucosidase 6-like | 240 | 0.0912 |
| Mechanosensitive ion channel | 358 | 0.1712 |
| Flap endonuclease GEN-like | 348 | 0.0959 |
| Lanthionine synthetase C-like | 348 | 0.1223 |
| Autophagy-related protein 13 | 254 | 0.1020 |
| Co/Zn/Cd efflux syst. component | 300 | 0.0453 |
| Auxin-induced protein PCNT115-like isoform | 1,309 | 0.1030 |
| Isocitrate/isopropylmalate DH | 354 | 0.0349 |
| Pumilio homolog 2-like | 241 | 0.0708 |
| Amino acid permeases | 391 | 0.0632 |
| Calmodulin-binding transcription activator | 1,358 | 0.0881 |
| Histidine kinase-like ATPases | 359 | 0.0228 |
| Bulb-type mannose-specific lectin | 405 | 0.0855 |
| S-adenosyl-methionine-sterol-C-methyltransferase | 404 | 0.0435 |
| Put efflux protein, MATE family | 402 | 0.0636 |
| AUX1-like permease | 404 | 0.0658 |
| Mean | 332.3 | 0.0770 |
